# Associations between successful palliative cancer pathways and community nurse involvement

**DOI:** 10.1186/1472-684X-8-18

**Published:** 2009-12-14

**Authors:** Mette Asbjoern Neergaard, Peter Vedsted, Frede Olesen, Ineta Sokolowski, Anders Bonde Jensen, Jens Sondergaard

**Affiliations:** 1 The Palliative Specialist Team, Department of Oncology, Aarhus University Hospital, Noerrebrogade, Aarhus, Denmark; 2 The Research Unit for General Practice, University of Aarhus, Bartholin Allé, Aarhus, Denmark; 3 Department of Oncology, Aarhus University Hospital, Randersvej, Aarhus, Denmark; 4 The Research Unit and Department for General Practice, University of Southern Denmark, J.B. Winsløws Vej, Odense, Denmark

## Abstract

**Background:**

Most terminally ill cancer patients and their relatives wish that the patient dies at home. Community nurses (CNs) are often frontline workers in the patients' homes and CN involvement may be important in attaining successful palliative pathways at home.

The aim of the present study was to examine associations between bereaved relatives' evaluation of palliative treatment at home and 1) place of death and 2) CN involvement.

**Methods:**

The study is a population-based, cross-sectional combined register and questionnaire study performed in Aarhus County, Denmark. CN questionnaires were used to obtain data on CNs' efforts, GP-questionnaires were used to obtain data on pathway characteristics and relatives answered questionnaires to evaluate the palliative pathway at home. Questionnaires addressed the palliative pathway of a total of 599 deceased cancer patients. Associations between bereaved relatives' evaluation of palliative pathways at home and place of death and CN involvement were analysed.

**Results:**

'A successful palliative pathway at home' was positively associated with home-death and death at a nursing home compared with death at an institution. No significant associations were identified between the evaluations of the palliative pathway at home and the involvement of CNs.

**Conclusions:**

Our study indicates that dying at home is positively associated with a higher likelihood that the bereaved relative will evaluate the palliative pathway at home as successful. The absence of any significance of involvement of CNs may be ascribed to the variables for involvement chosen in the study. More research is needed on CNs' impact on palliative pathways.

## Background

Most terminally ill cancer patients and their relatives wish that the patient be cared for and die at home [1-3] and home-death is therefore almost always the main outcome when evaluating end-of-life care. However, home-death is not necessarily tantamount to a successful palliative pathway at home, since many factors and the professionals involved can shape the patients' or the relatives' views. Furthermore, in a previous study we found that even though patients died at institutions (hospital and hospice), they spent three quarters of the time in the palliative pathway at home (mean, % (95% CI): 75.1 (71.0;79.1)) [4]. This makes it even more important to question home-death as a measure of a successful palliative period at home, i.e. the last period of the patient's life during which all curative treatment had been discontinued and care and treatment were provided for palliative purposes only.

The community nurses (CNs) play an important role during the palliative period when the patient is at home, since CNs are frontline workers whether a specialist team or only the GP is involved. Previous studies suggest that 24-hour back-up and overall involvement of the CNs is an important factor in bereaved relatives' evaluation of palliative pathways [5-7]. Furthermore, research has also shown that involvement of a CN is positively associated with home-death [4,8-10]. However, we lack sufficient knowledge of the importance of the CNs' personal data, skills and different services in relation to achieving a successful palliative pathway at home.

The aim of the present study was to examine the association between the bereaved relatives' evaluation of palliative pathways at home and 1) place of death and 2) CNs' involvement.

## Methods

**Figure 1:**
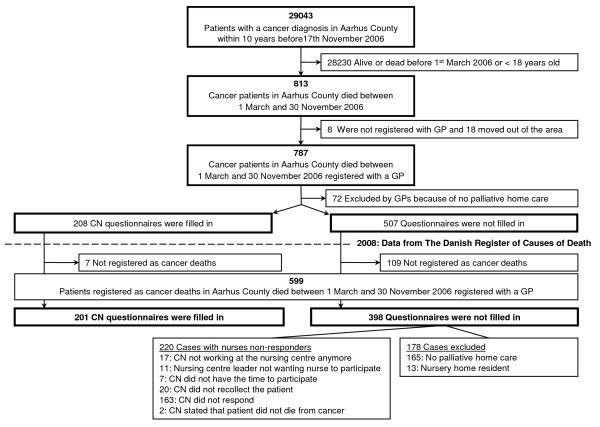
**Flow-chart of sampling of study population and data collection**. Community nurses (CNs) questionnaires. Responders and non-responders.

We conducted a population-based, cross-sectional combined register and questionnaire study. Questionnaires were filled in by relatives evaluating the palliative pathways, by CNs and by GPs. Questionnaires addressed the palliative pathways of a total of 599 deceased cancer patients (Figure 1).

### Setting

The Danish health care system is tax-financed and provides free care for those in need of help at home. Care is provided by GPs, CNs and home carers. CNs employed by the municipalities are most often involved in palliative pathways in the patient's home, especially in the terminal phase. They may visit patients on a 24-hour basis.

Denmark has no formal national agreement on task distribution in palliative care. Palliative specialist outgoing teams based at the major hospitals are available during daytime hours, and specialist advice can be obtained from these specialist teams by CNs or GPs by telephone.

The study took place in 2007 (January - July) in The Aarhus County, which at the time comprised approximately 640 000 inhabitants, 12% of the Danish population, 43 municipalities. The annual cancer mortality amounted to 1680 persons in 2005 [11]. Numbers for 2006 are still not available.

In Denmark, all citizens are registered with unique civil registration numbers [12]. Questionnaire data were linked to health register information by means of these numbers.

### Study population and sampling

Since no database on palliative patients is available in Denmark, we decided to focus on cancer patients since these patients are included in validated Danish registers. We sampled the patients by combining official register data with questionnaires information. The study included adults in Aarhus County who died from cancer from 1^st ^March to 30^th ^November 2006 and who had received some palliative home care either from CNs, GPs or a palliative specialist team.

From the county hospital discharge register, we identified 29,043 individuals above 18 years who were registered with at least one cancer diagnosis (ICD-10) (excluding non-melanoma skin cancers) during the period November 2006 and ten years back. In December 2006, using the Civil Registration System database, we identified 813 patients among the 29,042 who died from 1^st ^March to 30^th ^November 2006 (nine months). From the regional health authority's register, we identified their GPs. Eight (1.0%) patients were not registered with a GP and 18 (2.2%) had moved from the county after having been diagnosed, leaving 787 deceased cancer patients for analysis. A CN questionnaire was sent to the local health centre of the patient's address, and a GP questionnaire was sent to the patient's GP. They were asked about cause of death, if palliative care had been provided in the patient's home and if the closest bereaved relative could receive a questionnaire. If one of the two (CN or GP) advised against sending the relative a questionnaire, it was not sent. The closest relative was defined by the professionals, but if they stated that we could contact the relative, but without providing the name or the address, we sent the questionnaire to relatives in the following order: spouse, child above 18 years, oldest sibling and parent (data from the Civil Registration System database). We asked the person contacted to give the questionnaire to the closest relative involved in the palliative pathway.

In late 2008, data from the Danish Register of Causes of Death for deaths in 2006 were available. Merging the information of cancer deaths with our database, we excluded 188 patients who were not registered with cancer as cause of death, which reduced our study population to 599 deceased cancer patients (Figure 1).

### Data collection

The study comprises data from three mail-delivered questionnaires, one for CNs, one for GPs and one for relatives of deceased cancer patients. The questionnaires included themes identified through literature studies, clinical experience and group interview studies with bereaved relatives [5] and health professionals (CNs, GPs and hospital consultants). The themes in the questionnaires can be seen in Table 1. Only a small part of the data from the questionnaires was used in this study since it is part of a larger research project with present and future publications [13].

**Table 1:** Themes in questionnaires

CN questionnaire	Validation questions about the cause of death
(46-item questionnaire)	Information about closest relative and the CN/centre involved.
	CN knowledge of patient before the palliative pathway
	Type of contact
	Patient's wish for and actual place of death
	Care for the relative during the palliative period and after bereavement
	Cooperation with GPs, hospital doctors, palliative specialist team
	When discharging the patient from the hospital
	When the patient is at home
	Evaluation the primary care sector's effort
	Evaluation of CN's own effort
	Overall view on palliative care in primary care
	Demographic data of the CN
	Open question on comments to the questionnaire
**GP questionnaire**	Validation questions about the cause of death
(72-item questionnaire)	Information about closest relative and the community nurse/centre involved.
	GP knowledge of patient before the palliative pathway
	Length of the palliative period
	Type of contact
	Patient's wish for and actual place of death
	Care for the relative during the palliative period and after bereavement
	Cooperation with CNs, hospital doctors, palliative specialist team
	Especially when discharging the patient from the hospital
	When the patient is at home
	Evaluation the primary care sector's effort
	Evaluation of GP's own effort
	Overall view on palliative care in primary care
	Demographic data of the GP's practice
	Open question on comments to the questionnaire

**Questionnaire for bereaved relatives**	Demographic data of deceased
(65-item questionnaire)	Length of the palliative period
	Type of contact to professionals
	Patient's wish for and actual place of death
	Care for the relative during the palliative period and after bereavement
	Cooperation among GPs, CNs, hospital doctors, palliative specialist team
	When discharging the patient from the hospital
	When the patient is at home
	Evaluation the primary care sector's effort
	Demographic data of the relative
	Open question on comments to the questionnaire

The 46-item CN questionnaire was pilot-tested among 14 CNs who had also participated in a prior interview study[5]. Data included information on the nurse's age (-39, 40+), number of years as CN (0-5, 6+), amount of extra education or classes in palliative care (no, yes), knowledge of the patient before the palliative period (dichotomized into poor (1,2 on a 1-5-point scale) and good (3, 4 and 5)), whether the nurse had contact to the patient's relatives (no/yes) and how often the nurse or community services paid the patient a home-visit (less than once a day, once a day or more). If the CNs did not respond, two reminders were sent four and seven weeks after the first questionnaire, respectively.

The 72-item GP questionnaire was pilot-tested among 30 GPs in another Danish county. In this study, GP questionnaires were mainly used to locate relatives and CNs, to inform that a palliative pathway had taken place at home or not, and to determine the duration of the palliative period. Thus, data from the GP questionnaires included information on the involvement of a GP (no, yes) or a specialist team (no, yes) and the duration of the palliative period at home in weeks. The palliative period was defined as the last period of the patient's life during which all curative treatment had been discontinued and care and treatment were provided for palliative purposes only. GPs received a small economic compensation for their efforts since the GPs in Denmark only get a small regular income per patient registered with the GP and are mainly paid by the services performed. CNs, on the other hand, have a set income and received no compensation for participation. GP non-responders were sent reminders four and seven weeks following the first questionnaire.

The 65-item questionnaire to relatives was pilot-tested among 14 bereaved relatives not included in this study. Non-responders of relatives were sent one reminder four weeks after the first questionnaire. From the relatives we obtained data on their age (18-65, 66+), gender, relation to the deceased (non-spouse, spouse), whether they were living with the patient (no, yes) and their own vocational educational level (3 years or less, > 3 years). Furthermore, to examine 'A successful palliative pathway at home' the relatives were asked to evaluate the palliative pathway, answering the following question: 'How, in your own words, was the entire period at home during which the deceased was dying compared with how you felt it should have been?' (dichotomized into unsuccessful ('Fairly well', 'Bad', 'Very bad') and successful ('Very well', 'Well')).

We retrieved register data on patient age (18-65, 66+), gender, cancer diagnosis (lung, colo-rectal, breast, prostate, other) and place of death (institution (hospital and hospice), nursing home, home and other).

### Analysis

'A successful palliative pathway at home' as defined by the bereaved relative was used as the outcome measure and we calculated associations with 1) place of death and 2) nurse involvement. The multivariate model consisted of the variables seen in Table 2 plus the duration of the palliative period spent at home (number of weeks as a categorical variable), since it could be associated with the CNs' possibility to provide palliative care.

**Table 2:** Associations between a successful palliative pathway at home and model variables.

	Unadjusted prevalence ratio (95% CI)	Adjusted prevalence ratio (95% CI)
**Gender of relative**		
Male	1	1
Female	1.0 (0.8;1.4)	1.1 (0.7;1.7)

**Age of relative**		
18-64	1	1
65+	1.3 (1.0;1.7)	1.0 (0.8;1.6)

**Living with patient**		*Not included because of collinarity with*
No	1	*'Relatives' relation to deceased'*
Yes	1.6 (1.0;2.6)	

**Relative's relation to diseased**		
Not spouse	1	1
Spouse	1.4 (1.0;2.0)	0.9 (0.6;1.5)

**Relative's vocational education**		
3 years or less	1	1
> 3 years	0.8 (0.6;1.1)	0.7 (0.3;1.3)

**CN's age**		*Not included because of collinarity with*
Less than 40	1	*'CN's years as CN'*
40 and above	1.1 (0.8;1.4)	

**CN's years as CN**		
Five and less	1	1
More than five	1.2 (0.9;1.5)	0.9 (0.6;1.3)

**CN's extra education or courses in palliative care **(n (%))		
No	1	1
Yes	0.7 (0.5;1.2)	0.8 (0.4;1.7)

**CN's knowledge prior to palliative period**		
Poor	1	1
Well	1.2 (0.9;1.5)	1.3 (0.9;1.8)

**CN's contact with relatives**		
No	1	1
Yes	0.6 (0.6;0.7)	1.1 (0.7;1.8)

**CN's and community services' home-visits pr day**		
Less than one	1	1
One or more	0.9 (0.6;1.2)	0.7 (0.4;1.1)

**GP involvement**		
No	1	1
Yes	1.0 (0.7;1.3)	1.0 (0.5;1.7)

**Specialist team involvement**		
No	1	1
Yes	0.9 (0.7;1.3)	1.0 (0.7;1.4)

**Place of death**		
Institution (Hospital or Hospice)	1	1
Nursing home	1.3 (0.7;2.5)	1.8 (0.9;3.7)
Home	1.7 (1.1;2.7)	2.3 (1.2;4.4)

A total of 101 cases were included in the analyses. The unadjusted and the adjusted prevalence ratios (PRs) are shown with 95% confidence intervals (95%CIs). CN is the Community nurse involved.

Unadjusted and adjusted associations were calculated. Using robust variance estimates, the estimates were adjusted for clustering of patients seen by the same CN [14]. Prevalence ratios (PRs) with 95% confidence intervals (95% CIs) were used as a measure of association. Due to the high prevalence of the outcome measure (more than 20% 'successful palliative pathways at home'), odds ratios would overestimate the association [15,16]. PRs were calculated with generalised linear models (GLM) with log link and the Bernoulli family, and when the model did not converge, we used the Poisson regression model [15,17].

The variables were assessed for *collinearity *(Pearson's correlation coefficient > 0.4) and *multicollinearity *(variance inflation factor < 10) [18,19]. Due to collinearity, 'Relatives living with the patient' and 'CNs' age' were not included in the multivariate model. Neither forward nor backward selection was performed. Data were analyzed using STATA 10 [20].

### Ethics

According to Scientific Committee for the County of Aarhus, the Biomedical Research Ethics Committee System Act does not apply here. The study was approved by the Danish Data Protection Agency and the Danish National Board of Health.

## Results

**Figure 2:**
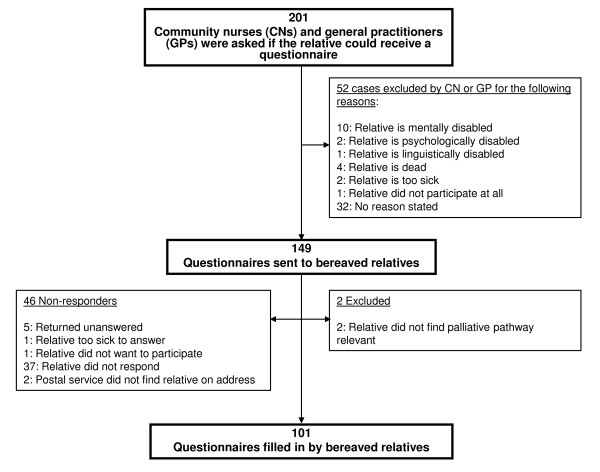
**Flow-chart of questionnaires to bereaved relatives**. Responders and non-responders.

A total of 201 questionnaires from 129 CNs were obtained. For 178 cases, the CN or GP stated that there had been no home care during the palliative pathway or that the patient had been a nursing home resident from the beginning of the palliative pathway and therefore had had no contact with CNs. The remaining cases all had a palliative period at home no matter where they eventually died. For 220 cases the nurse did not respond, leaving a response rate of 47.7% (Figure 2). The 220 cases from non-responding CNs were not statistically significantly different from the included cases in terms of patient gender and number of GP home-visits, but the non-responder cases tended to be significantly older and to die more often at a nursing home (Table 3).

**Table 3:** Characteristics of 201 included cases and 220 cases not included because the community nurses (CNs) did not respond.

	Cases of community nurse responders (N = 201, 129 nurses)	Cases of Community nurse non-responders (N = 220)
**CN's gender **(n (%))		**-**
Male	0 (0.0)	
Female	129 (100.0)	

**CN's age (**Mean (95% CI)**)**	45.3 (43.9;46.7)	-

**Years as nurse (**Mean (95% CI))	19.9 (18.2;21.5)	-

**Years as CN (**Mean (95% CI))	10.7 (9.5;12.0)	-

**Number of questionnaires pr CN (**Median (IQI))	1.4 (1.3;1.5)	-

**CN's extra education or courses in palliative care **(n (%))		-
No	107 (84.9)	
Yes	19 (15.1)	

**Patient's age at time of death **(mean (95% CI))*	70.0 (68.4;71.6)	72.8 (71.0;74.5)

**Patient's gender **(n (%))		
Male	111 (55.2)	129 (58.4)
Female	90 (44.8)	91 (41.6)

**Primary cancer diagnosis **(n (%))		
Bronchus/lung	34 (16.9)	48 (21.8)
Colon/rectum	26 (12.9)	39 (17.7)
Breast	23 (11.4)	20 (9.1)
Prostate	31 (15.4)	26 (11.8)
Other	87 (43.4)	87 (39.6)

**Place of death **(n (%))*		
Home	97 (48.3)	79 (35.9)
Nursing home	35 (17.4)	59 (26.8)
Hospital/hospice	67 (33.3)	82 (35.9)
Other (e.g. other institution)	2 (1.0)	3 (1.4)

* Statistically significantly different from 101 cases included in study with p-value < 0.05

Data on nurses obtained from CN questionnaires. Case data are obtained from formal health registers.

For 52 cases (25.9% of the 201 cases with nurse response), the CN or GP advised against sending the relative a questionnaire (Figure 2). A total of 101 relative questionnaires were obtained and two were excluded since the relative stated that the questionnaire was not relevant to the pathway at all, leaving 46 relatives as non-responders (response rate 68.7%) (Figure 2).

Comparison of the 101 included cases with the 46 cases where the relatives did not respond showed that relatives answered the questionnaire significantly more frequently when the questionnaire concerned a male patient (p-value < 0.05) than when it concerned a female patient (Table 4).

**Table 4:** Characteristics of 101 included cases, the 46 cases not included because the relative did not respond and the 52 cases where the general practitioners (GP) or the community nurse (CN) advised against sending the relative a questionnaire.

	Cases in study (N= 101)	Cases of relative-non-responders (N = 46)	Cases where the GP or CN advised against sending the relative a questionnaire (N = 52)
**Patient's age at time of death** (mean (95% CI))	69.6 (67.3;71.9)	68.1 (64.8;71.4)	73.0 (69.9;76.2)

**Patient's gender **(n (%))			
Male	61 (60.4)	18 (39.1)*	30 (57.7)
Female	40 (39.6)	28 (60.9)*	22 (42.3)

**Primary cancer diagnosis **(n (%))			
Bronchus/lung	16 (15.8)	10 (21.7)	8 (15.4)
Colon/Rectum	13 (12.9)	8 (17.4)	5 (9.6)
Breast	11 (10.9)	9 (19.6)	3 (5.8)
Prostate	17 (16.8)	3 (6.5)	11 (21.2)
Other	44 (43.6)	16 (34.8)	25 (48.1)

**Place of death (**n (%))			
Home	62 (61.4)	25 (54.4)	10 (19.2)*
Nursing home	13 (12.9)	3 (6.5)	18 (34.6)*
Hospital/Hospice	25 (24.8)	18 (39.1)	23 (44.2)*
Other (e.g. other institution)	1 (1.0)	0 (0.0)	1 (1.9)

**Relative's age at time of filling in questionnaire**		-	-
(mean (95% CI))	58.3 (55.4; 61.2)		

**Gender of relative **(n (%))		-	-
Male	28 (27.7)		
Female	73 (72.3)		

**Relative's relation to deceased **(n (%))		-	-
Spouse	63 (62.4)		
Girlfriend or boyfriend	1 (1.0)		
Daughter or son	32 (31.7)		
Sister or brother	1 (1.0)		
Parent	1 (1.0)		
Daughter-in-law	3 (3.0)		

**Relative lived with patient **(n (%))		-	-
No	23 (23.7)		
Yes	74 (76.3)		

**Relative's vocational education **(n (%))		-	-
3 years or less	62 (63.3)		
> 3 years	36 (36.7)		

**CN's age **(mean (95% CI)	44.4 (42.7;46.2)	46.2 (43.8;48.6)	46.1 (43.9;48.4)

**CN's years as CN **(n (%))			
Five and less	36 (40.0)	16 (40.0)	12 (25.0)
More than five	54 (60.0)	24 (60.0)	36 (75.0)

**CN's extra education or courses in palliative care **(n (%))			
No			
Yes	70 (82.4)	34 (85.0)	39 (86.7)
	15 (17.6)	6 (15.0)	6 (13.3)

**CN's and community services' home-visits pr day **(n (%))			
Less than one			
One or more	14 (14.7)	5 (12.2)	9 (18.4)
	81 (85.3)	36 (87.8)	40 (81.6)

**CN's knowledge prior to palliative period **(n (%))			
Poor			
Well	57 (60.6)	22 (51.2)	29 (56.9)
	37 (39.4)	21 (48.8)	22 (43.1)

**CN's contact with relatives **(n (%))			
No	3 (3.1)	2 (4.7)	15 (30.6)*
Yes	94 (96.9)	41 (95.3)	34 (69.4)*

**GP involvement **(n (%))			
No	14 (15.2)	4 (9.3)	10 (20.0)
Yes	78 (84.8)	39 (90.7)	40 (80.0)

**Specialist team involvement **(n (%))			
No	39 (55.7)	21 (58.3)	21 (56.8)
Yes	31 (44.3)	15 (41.7)	16 (43.2)

* Statistically significantly different from the 101 cases in study with p-value < 0.05.

Not all sums of percentages are added to 100.0% because of round-offs.

Case data in study stem from GP, CN and relative-questionnaires and from formal health registers. Case data of relative-non-responders and of the group where the GP advised against sending the relative a questionnaire stem from GP and CN questionnaires and formal health registers.

Comparing the 101 included cases with the 52 cases where CNs or GPs advised against sending the relative a questionnaire showed that cases included comprised more home-deaths, less institutional and nursing-home-deaths and were characterised by the CNs having more contact to the relatives (p-value < 0.05) (Table 4).

### Associations with evaluation of palliative pathway at home

'A successful palliative pathway at home' was statistically significantly associated with home-death (2.3 (95% CI: 1.2;4.4)) (Table 2). It was also associated with nursing home-death compared with hospital-death (1.8 (95% CI: 0.9;3.7), even if the association fell short of significance. None of the variables concerning CN involvement were statistically significantly associated with a successful palliative pathway at home.

## Discussion

### Main findings

In a group of patients who died from cancer and had a palliative pathway at home, we found that the relatives' positive evaluation of the palliative pathway at home was associated with home-death and nursing-home-death. However, the latter association was not significant. Surprisingly, we identified no significant associations between the evaluations and the involvement of CNs.

### Strengths and limitations of the study

The strengths of this study are its sampling and its comprehensive data collection. To eliminate differential misclassification, we used the standardised official health registers to identify the study population, including the places and the reasons of death. To minimise recall bias, the questionnaire was sent in January 2007. Thus, we did not await the update of the Danish Register of Causes of Death in 2008.

The major weakness of these analyses was the selection bias. We found differences between included cases and the cases where the relatives had been excluded by CNs or GPs. The differences meant that we would tend to exclude those cases where the CN was not as involved and did not know the patient as well as in those cases that were included. In these excluded cases, the relative may evaluate the pathway as less successful or tend to see the nurses' involvement as less important. Thus, we would tend to overestimate the associations between a successful pathway at home and CN-related variables. This would also be the case if the CNs or the GPs had excluded cases that they knew had been unsuccessful (which they might have chosen to do for a number of reasons) despite CN involvement. As home-death is associated with CN involvement [8-10], such selection bias, therefore, tends to strengthen the association between home-death and a successful pathway at home. Furthermore, it is seen from Table 4 that more relatives of male than of female patients answered the questionnaires compared to the non-responding group. However, there is no evidence that the patient's sex is associated with the relatives' satisfaction of end-of-life care [21].

Our results are generalizable to patients who receive palliative home-care in a healthcare system similar to the Danish system because we sampled patients who died from cancer and had some palliative period at home, regardless of the involvement of specialised teams or hospital records and because Denmark is quite homogenous with respect to primary care and social demography.

Approximately 1680 patients died from cancer in Aarhus County in 2006, and we included 599 cases recruited during a nine-month period (Figure 1). The discrepancy between the total number of deaths and the number of cases included may appear because we did not include persons less than 18 years, non-melanoma cancer cases, and because we included only those with a cancer diagnosis registered in a hospital in Aarhus County as their main diagnose of admittance within a 10-year period. Furthermore, the period where patients could die (1^st ^March-30^th ^November) did not include the winter months of 2006, which may imply that some cases were missing, since winter months may have a higher average of deaths than the rest of the year.

### Discussion of results and comparison with existing literature

We found that home-death was statistically significantly associated with the relatives' evaluation of the palliative pathway at home as being more successful compared with pathways ending with institutional death. In line with this, previous studies have shown that home-death was associated with better bereavement response [22-24] and overall satisfaction with the palliative pathway at home [21]. The fact that a successful palliative pathway at home is associated with home-death is hardly surprising. Most studies show that most terminally ill patients and their relatives wish death to take place at home [3,25-27] and an institutional death may be a direct consequence of a poor palliative pathway at home. However, one may also argue that in the last days of life, being at home may be distressing for the relatives, and an institutional death may be preferred even if the palliative pathway at home has, indeed, been successful. It may even make the pathway at home look even more successful in retrospect since the distressing and care-demanding last days of the patient's life did not take place at home. In this context, the found association is more interesting.

The association between a successful pathway at home and home-death in this study may also be partly rooted in the fact that patients dying at a hospital or hospice often have worse symptoms and problems, e.g. pain, than patients dying at home, which, indeed, would affect the relatives' evaluation. We included no variables that could describe symptom and problem severity since the included variables were identified through literature studies, clinical experience and group interview studies with bereaved relatives [5] and involved professionals (CNs, GPs and hospital consultants), and this issue did not come up. However, patients with severe symptoms may also have more contact with a palliative specialist team, and if we had adjusted for this we might have reduced some confounding. However, eliminating 'specialist team involvement' from the model would weaken the association between a successful pathway at home and home-death, but only slightly, which indicates that symptoms may be a confounder for which control was not fully achieved.

We also found that dying at a nursing home was associated with the relatives' evaluation of the palliative pathway at home compared with institutional death. But this association fell short of statistical significance in our study with only 101 included cases. A reason for this result could, again, be that patients dying at a hospital or hospice presumably have worse symptoms than those who die at home and at nursing homes. Further research is needed to investigate the implications of death at home and at nursing homes for patients and relatives.

Quality in dying is much debated and there is no doubt that what makes a good death is determined by a complex interplay of many factors, e.g. personal and cultural values, supportive network characteristics, physical and medical factors, and the services offered by the health care systems. Surprisingly, we found that none of the CN-related factors in our model were statistically significantly associated with the relatives' evaluation of the palliative pathway at home. To our knowledge, no previous studies have explored the association between relatives' or patients' evaluation of the palliative pathway at home and CN-related factors. However, studies show that the following factors are positively associated with bereaved relatives' satisfaction with the CN during the palliative pathway: frequent CN home-visits, visiting at night, knowing enough and spending enough time in the homes[28,29]. The lack of significance of CNs' involvement may be due to the involvement variables chosen in this study, or maybe the CNs' effect on the pathway at home may be like a package, where it is more a question of the existence of a CN-service in the home or not.

It is striking that we observed no association with the included variables in the model, except for the place of death. This may be due to the small size of the study and the amount of variables included, but it may also be due to the fact that other kinds of factors are pivotal to relatives in achieving a good palliative pathway at home, i.e. the relation between the patient and the relative; the back-up provided by other relatives and colleagues; patient factors; and factors relating to practical arrangements in the home, etc. This, again, calls for more research in the field.

### Implications for future research

More research is needed on how to measure bereaved relatives' evaluation of palliative pathways, how the factors concerning CNs' involvement are affecting this evaluation and what constitutes a well-performed palliative effort by the CNs. Furthermore, studies of the predictive power of a more active approach in primary health services in achieving a successful palliative pathway are needed.

## Conclusion

Our study indicates that dying at home is positively associated with an increase in the likelihood that the bereaved relative will evaluate the palliative pathway at home as successful. Symptom severity may be an important confounder; one that was not adjusted for in the present study. No significant associations between the evaluations and way the CNs were involved could be identified. There is a need for studies exploring predictors of the primary care effort associated with a "good death" to improve and ensure more focus in the palliative primary health care effort.

## Competing interests

The authors declare that they have no competing interests.

## Authors' contributions

MAN participated in the design of the study, in the development of the questionnaires, handled the data, drafted the manuscript and performed the statistical analysis.

PV helped to draft the manuscript and IS helped to perform the statistical analysis

FO, ABJ and JS participated in the design of the study and in the development of the questionnaires. JS also helped to draft the manuscript.

All authors read and approved the final manuscript.

## Pre-publication history

The pre-publication history for this paper can be accessed here:

http://www.biomedcentral.com/1472-684X/8/18/prepub
